# The effect of Shengmai injection in patients with coronary heart disease in real world and its personalized medicine research using machine learning techniques

**DOI:** 10.3389/fphar.2023.1208621

**Published:** 2023-09-14

**Authors:** Jing Ma, Ze Yu, Ting Chen, Ping Li, Yan Liu, Jihui Chen, Chunming Lyu, Xin Hao, Jinyuan Zhang, Shuang Wang, Fei Gao, Jian Zhang, Shuhong Bu

**Affiliations:** ^1^ Department of Pharmacy, Xinhua Hospital, Shanghai Jiaotong University School of Medicine, Shanghai, China; ^2^ Institute of Interdisciplinary Integrative Medicine Research, Shanghai University of Traditional Chinese Medicine, Shanghai, China; ^3^ Experiment Center for Science and Technology, Shanghai University of Traditional Chinese Medicine, Shanghai, China; ^4^ Dalian Medicinovo Technology Co., Ltd., Dalian, China; ^5^ Beijing Medicinovo Technology Co., Ltd., Beijing, China

**Keywords:** Shengmai, coronary heart disease, personalized medicine, machine learning, real world

## Abstract

**Objective:** Shengmai injection is a common treatment for coronary heart disease. The accurate dose regimen is important to maximize effectiveness and minimize adverse reactions. We aim to explore the effect of Shengmai injection in patients with coronary heart disease based on real-world data and establish a personalized medicine model using machine learning and deep learning techniques.

**Methods:** 211 patients were enrolled. The length of hospital stay was used to explore the effect of Shengmai injection in a case-control study. We applied propensity score matching to reduce bias and Wilcoxon rank sum test to compare results between the experimental group and the control group. Important variables influencing the dose regimen of Shengmai injection were screened by XGBoost. A personalized medicine model of Shengmai injection was established by XGBoost selected from nine algorithm models. SHapley Additive exPlanations and confusion matrix were used to interpret the results clinically.

**Results:** Patients using Shengmai injection had shorter length of hospital stay than those not using Shengmai injection (median 10.00 days vs. 11.00 days, *p* = 0.006). The personalized medicine model established via XGBoost shows accuracy = 0.81 and AUC = 0.87 in test cohort and accuracy = 0.84 and AUC = 0.84 in external verification. The important variables influencing the dose regimen of Shengmai injection include lipid-lowering drugs, platelet-lowering drugs, levels of GGT, hemoglobin, prealbumin, and cholesterol at admission. Finally, the personalized model shows precision = 75%, recall rate = 83% and F1-score = 79% for predicting 40 mg of Shengmai injection; and precision = 86%, recall rate = 79% and F1-score = 83% for predicting 60 mg of Shengmai injection.

**Conclusion:** This study provides evidence supporting the clinical effectiveness of Shengmai injection, and established its personalized medicine model, which may help clinicians make better decisions.

## 1 Introduction

Recently, the usage of Shengmai injection in treating cardiovascular diseases has raised concern, such as the treatment of hypertension and chronic heart failure (Wang et al., 2020a; Wang et al., 2020b; Zhang Y. et al., 2020; Cao et al., 2022). Shengmai injection is formed from Shengmai San by modern drug extraction methods, which increases the absorption rate and bioavailability of the active ingredients to achieve better efficacy (Wang et al., 2020b; Niu et al., 2022). Shengmai San is a traditional Chinese herbal prescription originated in the Jin dynasty. It is composed of Ginseng, Radix Ophiopogon, and Schisandra chinensis, whose active components are ginsenoside, Maidong saponin, and Schisandrin, respectively (Deng, 2011; Guo et al., 2015; Lu et al., 2021). Ginsenoside can improve circulation, adjust blood pressure, promote protein synthesis and ameliorate metabolism (Guo et al., 2015). The effect of Shengmai injection is determined by the interaction of these components. According to the theory of traditional Chinese medicine (TCM), Shengmai San is effective in replenishing Qi and nourishing Yin, recovering pulse, and stopping abnormal sweating (Chen et al., 2013; Wang et al., 2020b). The pharmacological mechanisms of Shengmai injection have been investigated. Shengmai injection can prevent myocardial calcium overload, alleviate myocardial hypertrophy, enhance myocardial contractility and protect endothelial function by protecting cardiomyocytes, reducing ischemia-reperfusion injury and cardiomyocyte apoptosis (Chen et al., 2013). Furthermore, Shengmai injection has been proven to inhibit local angiotensin II activity that contributes to the alleviation of ventricular hypertrophy, protect oxidative damage in mitochondria, cells, and tissues against oxidative damage, as well as improve hemodynamics parameter, increase sympathetic tone, enhance sinus node function, and improve conduction (Ding et al., 2007; Leong et al., 2010; Chen et al., 2013).

Some animal experiments have shown that Shengmai injection can reduce left ventricular mass and cardiac mass in rats with chronic heart failure, which indicates a positive effect on cardiac function and ventricular remodeling (Wang et al., 2020b). In addition, certain randomized controlled trials (RCTs) have found that compared with Western medicine alone therapy, adjuvant therapy of Shengmai injection leads to a higher reaction rate and greater improvements in cardiac function indicators (Wang et al., 2020b). Zhou Q, et al. reviewed some RCTs and found that treatment of Shengmai plus usual treatment can improve New York Heart Association (NYHA) functional classification than using usual treatment alone (Zheng et al., 2011). In addition, four RCTs indicated that treatment of Shengmai injection reduces the fatality rate in patients with acute myocardial infarction (Gao et al., 2008). However, the results of these previous RCTs are not consistent, possibly attributing to small sample sizes (Wang et al., 2020b). Moreover, because of local early drug marketing policy, many injections of TCM are lack of rigorous evaluations of effectiveness and safety, especially evidence from real-world study about the effectiveness of Shengmai injection on coronary heart disease were rare (Wang et al., 2020b). Herein, we tried to explore the clinical effect of Shengmai injection based on real-world data, in order to provide a high-quality research to guide the usage of Shengmai to clinicians.

Additionally, some adverse events during the treatment of Shengmai injection have been reported, such as allergic shock, nausea, bloating, and rash (Li et al., 2009; Guo et al., 2015). However, the causal relationship between these adverse events and Shengmai injection remains uncertain. For instance, ginsenoside was presumed to be the potential component that induces allergic shock, but the association with Shengmai injection was unclear (Guo et al., 2015). Some drugs’ doses can be affected on an individualized basis, such as age, body-mass index, comorbidities and other clinical parameters (Elemento, 2020). Inter-individual variation decides different drug effectiveness and adverse reactions, which necessitates the establishment of personalized medicine model. One important step of personalized medicine is to find factors that reflect drug response or adverse reactions. Therefore, we attempted to explore the important factors influencing dose of Shengmai injection and establish a personalized medicine model to achieve an optimum of medication effectiveness and safety.

With the development of machine learning and deep learning techniques, increasing studies have applied these techniques to establish personalized medicine models, which can enhance the model expression of complicated associations between individual factors and medication dose. Compared with Linear Regression models, machine learning and deep learning models can deal with real-world evidence with facility. It is because that machine learning and deep learning techniques can process complex, high-dimensional and interactive variable relationship, as well as these techniques can establish models with strong generalization and good accuracy (Kruppa et al., 2012; Lee et al., 2018; Mo et al., 2019). In recent years, some algorithms with more sophisticated principles have been developed, such as Gradient Boosting Decision Tree (GBDT), eXtreme Gradient Boosting (XGBoost), Categorical Boosting (CatBoost), Light Gradient Boosting Machine (LightGBM), and TabNet, which have been highly recognized in algorithm competitions (Chen et al., 2016; Ke et al., 2017; Prokhorenkova et al., 2017; Arik and Pfister, 2019; Janßen et al., 2019). With the increasing number of input sample data, machine learning and deep learning models can continually optimize parameters to refine model performance and practicality.

We aimed to explore the clinical effect of Shengmai injection in patients with coronary heart disease and establish a personalized medicine model using machine learning and deep learning techniques, in order to achieve a balance of medication effectiveness and safety.

## 2 Materials and methods

### 2.1 Study population

This is a retrospective study. Firstly, we conducted a case-control study to investigate the effects of Shengmai injection on patients with coronary heart disease. The main measurement was the length of hospital stay. At Xinhua Hospital affiliated to Shanghai Jiaotong University School of Medicine, patients who were diagnosed with coronary heart disease and were treated with Shengmai injection intravenously from 31 January 2018 to 14 September 2019 were enrolled in the experimental group. Those who were diagnosed with coronary heart disease but did not took Shengmai injection from 31 August 2015 to 26 February 2018 were enrolled in the control group. Secondly, based on the patient information in experimental group, who show effective and safety outcome after using Shengmai injection, a personalized medicine model was established using a machine learning or deep learning technique with an optimum predictive performance. Data in experimental group were divided into training cohort and test cohort according to a ratio of 8:2. An external verification cohort was used for model performance validation, enrolling patients diagnosed with coronary heart disease and treated with Shengmai injection intravenously from 1 October 2019 to 1 March 2020. All data in the original and external dataset were collected according to the same inclusion and exclusion criteria:

Including: 1) age >18 years; and 2) having coronary heart disease.

Excluding: 1) pregnant patients; and 2) patients with severe liver and/or renal injury.

Study data have been fully deidentified, and confidential information of patients has been deleted, in accordance with the CIOMS/WHO International Ethical Guidelines for Health-related Research Involving Humans (2016). Consequently, the study was deemed exempt from informed consent by study participants.

### 2.2 Data collection and processing

All data were collected from electronic medical records in the hospital information system, including medication information (days of using medication and length of hospital stay), demographic information (age and gender), underlying diseases [basic diseases (smoking history, drinking history, hypertension, diabetes, and hepatitis), coronary heart disease, pulmonary infection, cerebral infarction, atrial fibrillation, and myocardial infarction], drug combination (lipid-lowering drugs and platelet-lowering drugs), and assay index (blood routine test, urine routine test, blood biochemistry test, coagulation test, and tumor index at admission). The target variable was the daily dose of Shengmai injection, including daily dose of 40 and 60 mg.

**FIGURE 1:**
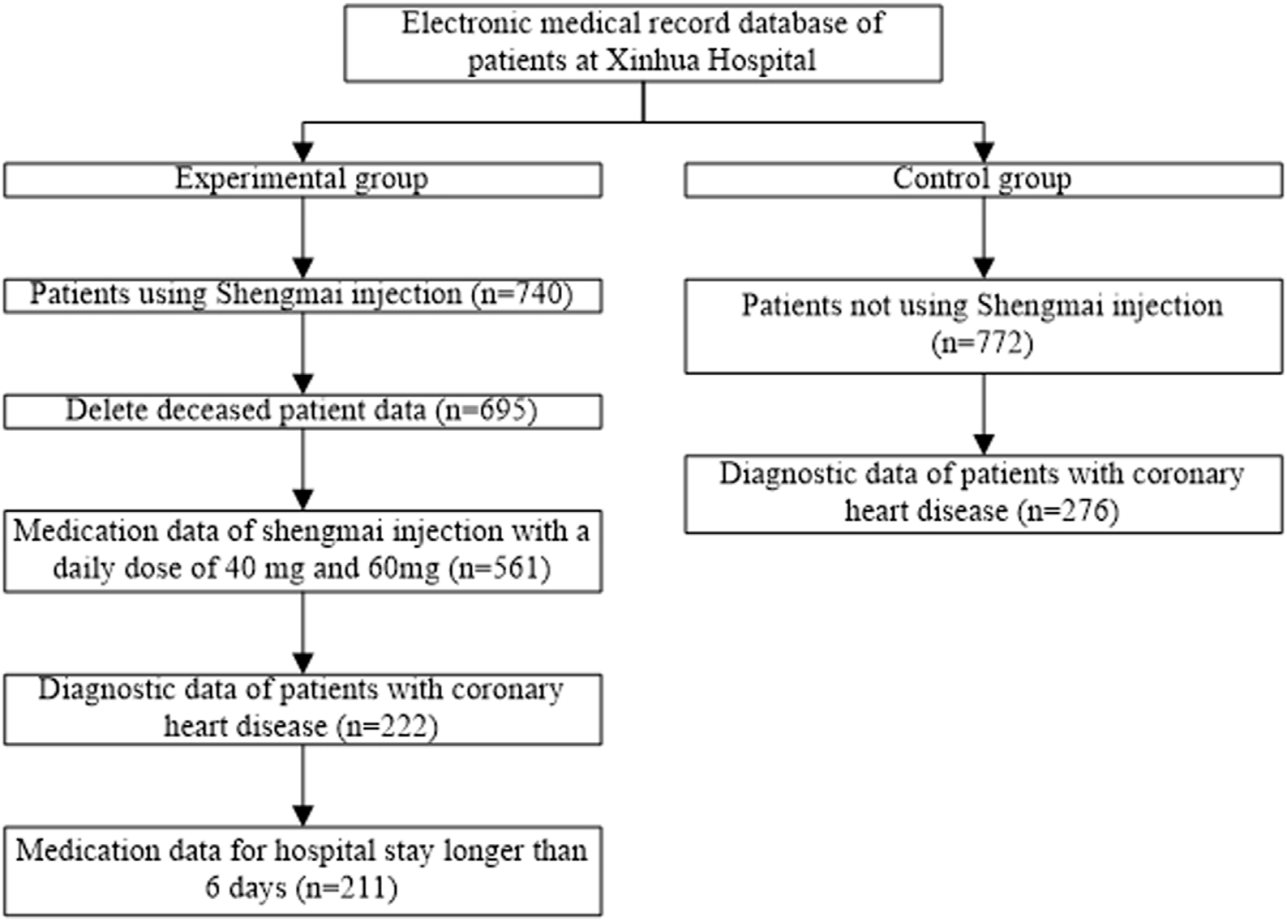
Workflow of data cleaning.

The workflow of data cleaning was displayed in Figure 1. The medication information of using Shengmai injection were extracted from medical records, and 740 cases were obtained after deleting the missing value of patients’ medication data. After deleting the death data, 695 cases remained. Subsequently, data of 561 cases were obtained by extracting Shengmai medication information with daily dose of 40 and 60 mg (daily dose = frequency of administration * single dose). Meanwhile, the diagnostic information of patients was extracted from medical records, mainly the data of patients with coronary heart disease and myocardial infarction. After combining the information of medication and disease diagnosis, 222 cases were obtained. After combining the information of medication and hospitalization, 211 cases were obtained by deleting the cases with hospital stay less than 6 days. If the patient’s admission time was empty, the start time of medication should be taken as the admission time; if the patient’s discharge time was empty, the end time of medication should be regarded as the discharge time, and the length of hospital stay was calculated. Herein, we divided the medication data into high and low dose groups. Daily dose of 40 mg Shengmai injection was considered as low-dose group, labeled “0”, which contained 95 cases. Daily dose of 60 mg Shengmai injection was regarded as the high-dose group, labeled “1”, which contained 116 cases. 51 variables were obtained after deleting variables with missing values of more than 50% and classification imbalance in medication data. Ultimately, among the 211 cases, demographic information, medical history, combination medication, and assay index were collected. Next step was to process control group data. The medication information of patients, who did not use Shengmai injection, was extracted from medical records, and 772 cases were obtained. After extracting the data of patients with coronary heart disease and myocardial infarction as well as deleting the missing value of more than 50% data, 276 cases were obtained. A total of 58 cases were included in the external verification cohort. Then the demographic information, medical history, combination medication, and assay index were collected.

### 2.3 Analysis of clinical effect

In the analysis of clinical effect, we tested the hypothesis that patients with coronary heart disease using Shengmai injection could have more effective outcome than in those not using Shengmai injection, and the length of hospital stay was set as the main measurement. Firstly, propensity score matching (PSM) was applied to process patient data. PSM referred to the screening of the experimental group and the control group by certain statistical methods, thus the screened subjects were comparable in clinical characteristics, meaning that the baseline levels of patients were similar. PSM can reduce the effects of selection bias and potential confounding factors (Glynn et al., 2006). In this study, the experimental and control groups were matched in a ratio of 1:1. It means that each two experimental groups matched one control group with the most similar propensity score. At last, if there was a difference in the outcome between the experimental group and the control group, it can be completely attributed to experimental factors.

After PSM, normality test was conducted for the length of hospital stay between the experimental group and the control group. If the data distribution was normal, independent *t*-test was used. If the data distribution did not comply with the normal distribution, Wilcoxon rank sum test was used to compare the length of hospital stay between the experimental group and the control group to determine whether there was a statistical difference, *p*-value ≤0.05 was considered significant.

### 2.4 Variable selection

Before modelling, a univariate correlation testing procedure was implemented to reduce the interference of irrelevant variables to the dose prediction model of Shengmai injection. Chi-square test was used for categorical variables, and Mann-Whitney U test was applied for continuous variables by investigating the association between the variable and outcome, *p*-value ≤0.05 was considered significant.

Subsequently, the univariates were further screened by machine learning. Sequential Feature Selection (SFS) based on the algorithm with the best predictive ability was applied to select certain variables to reach the optimum accuracy. The SFS algorithm added one feature to the feature subset each time, iteratively generated a new model, and calculated the model performance (accuracy) (Hatamikia et al., 2014). The iteration stopped when the accuracy of the feature subset reached the optimal value. The feature subset with the minimum size and optimum accuracy was thereby selected.

In order to ensure data integrity and create maximum use of existing Shengmai injection data, it is necessary to implement interpolation for missing values of the selected variables. RF model had high accuracy, a certain anti-noise ability, strong adaptability to discrete and continuous data, and hardly appeared over-fitting (Shah et al., 2014). Therefore, the missing values were interpolated based on the RF algorithm.

### 2.5 Model establishment

**FIGURE 2:**
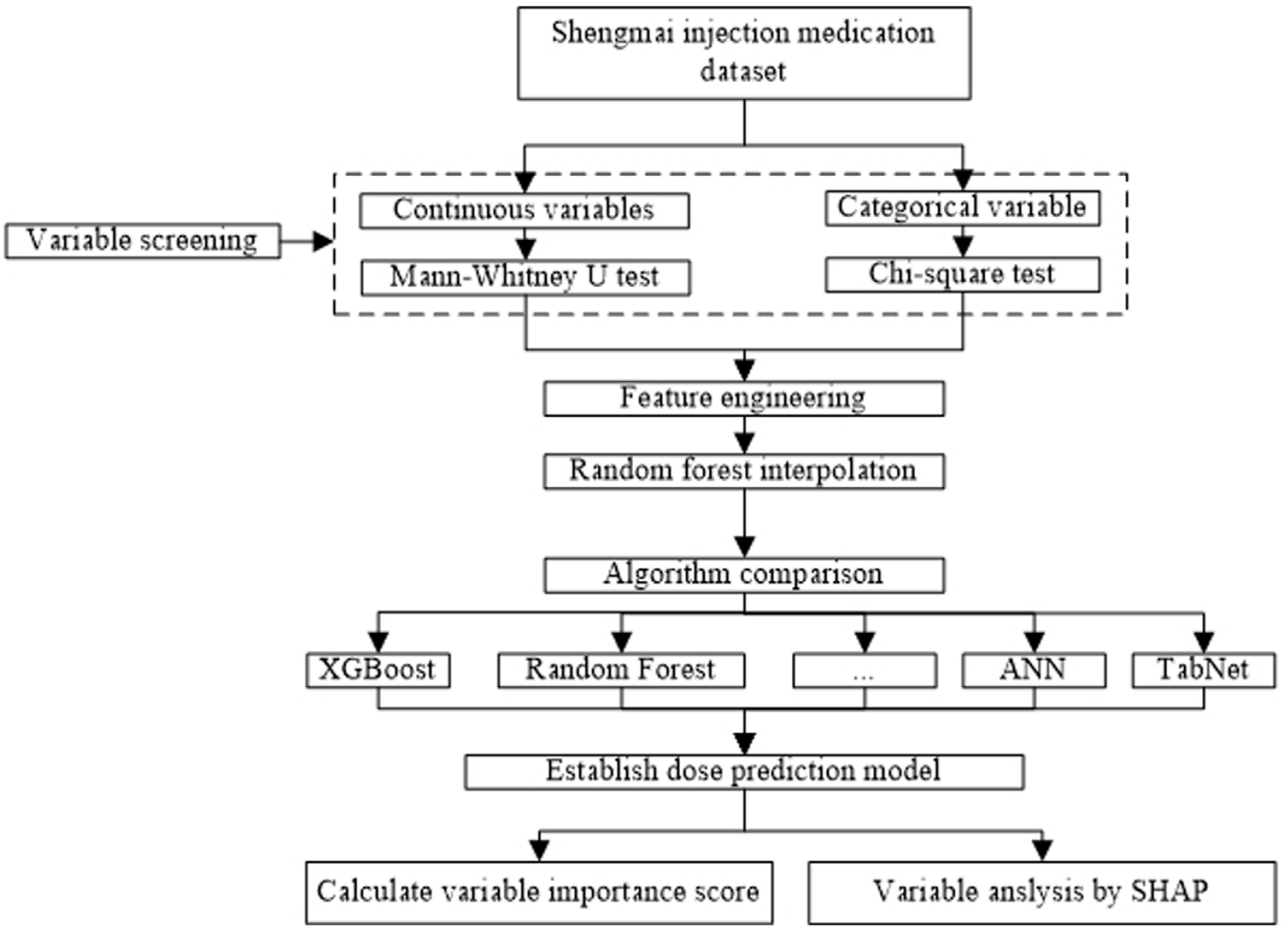
Workflow of data processing and modelling.

TP: true positive, indicating the positive class is predicted as the number of positive classes; TN: true negative, indicating the negative class is predicted as the number of negative classes; FP: false positive, indicating the negative class is predicted as the number of positive classes; FN: false negative, indicating the positive class is predicted as the number of negative classes.

In this study, the daily dose of Shengmai injection was set as the target variable, and daily dose of 40 mg corresponds to “0” and daily dose of 60 mg corresponds to “1”. Models were established on the training cohort, and the prediction performance of different models was calculated on the test cohort after parameter tuning. The modelling process was illustrated in Figure 2. The dose prediction model was established and compared by nine algorithms, including XGBoost, LightGBM, CatBoost, RF, GBDT, Support Vector Regression (SVR), Logistic Regression (LR), Artificial Neural Network (ANN) and TabNet, respectively. The dose prediction performance of all models was evaluated through precision, recall, F1-score, accuracy, and area under the curve (AUC). F1_score was used to measure the merits and defects of the model, higher F1_score indicating better model performance. Cross validation was applied to evaluate model generalization. Ultimately, model with the best evaluating indexes was selected as the final model to predict the dose of Shengmai injection.

$$\text{Accuracy}=\left(\text{TP}+\text{TN}\right)\;/\;\left(\text{TP}+\text{FN}+\text{FP}+\text{TN}\right)$$

$$\text{Precision}=\text{TP }/\;\left(\text{TP}+\text{FP}\right)$$

$$\text{Recall}=\text{TP }/\;\left(\text{TP}+\text{FN}\right)$$

$$F1\_\text{score}=2\times \text{TP }/\;\left(2\times \text{TP}+\text{FP}+\text{FN}\right)$$

### 2.6 Clinical interpretation

The importance scores of the selected variables above were calculated and ranked. The importance score of variable refers to the degree to which each variable in the model contributes to improving the predictive power of the whole model. Herein, we used the algorithm with the best model performance to calculate and rank the variable importance scores. Those ranked at the top of the list were selected as the important variables to construct model. Variables with higher importance scores were more closely related to the accurate prediction of Shengmai dose. Afterward, we used the SHapley Additive exPlanations (SHAP) to visually interpret the impacts of important variables on the model output (Lundberg and Lee, 2017). SHAP could help to explain which variables had positive or negative impacts on predicting Shengmai dose. One dot represents one sample. When the dot color is redder, the SHAP value gets larger; when the dot color is bluer, the SHAP value gets smaller. For a variable, when the majority of red dots appear in the region where the SHAP values are greater than 0, meanwhile, the majority of blue dots appear in the region where the SHAP values are less than 0, indicating that this variable has positive impact on the daily dose of Shengmai injection. Eventually, confusion matrix was used to analyze the model performance in test cohort and calculate the prediction precision.

## 3 Results

### 3.1 Baseline information

The baseline information of 211 patients in the experimental group was displayed in Table 1. The percentage of patients using the daily dose of 40 mg Shengmai injection occupied 45.02% and 54.98% for those using the daily dose of 60 mg Shengmai injection. The median [interquartile range (IQR)] medication days was 6.00 (4.00–8.00) days and the median (IQR) length of hospital stay was 11.00 (9.00–16.00) days. The median (IQR) patient age was 70.00 (63.00–81.00) years and male patients occupied 64.93%. The percentage of patients with basic diseases, coronary heart disease, pulmonary infection, cerebral infarction, atrial fibrillation, and myocardial infarction account for 79.62%, 92.94%, 19.91%, 13.27%, 20.38%, and 45.97%, respectively. For drug combination, patients using lipid-lowering drugs occupied 40.28% and those using platelet-lowering drugs occupied 30.81%.

**TABLE 1:** Baseline information.

Categories	Variables			Cases (N = 211)	Missing rate (%)
Target variable	Daily dose of Shengmai injection, mg, median (IQR)		40	95 (45.02%)	0
			60	116 (54.98%)	0
Medication information	Medication days, d, median (IQR)			6.00 (4.00–8.00)	0
	Length of hospital stay, d, median (IQR)			11.00 (9.00–16.00)	0
Demographic information	Age, y, median (IQR)			70.00 (63.00–81.00)	0
	Gender, n (%)	Male		137 (64.93%)	0
		Female		74 (35.07%)	0
Basic disease	Basic disease, n (%)			168 (79.62%)	0
	Coronary heart disease, n (%)			195 (92.42%)	0
	Pulmonary infection, n (%)			42 (19.91%)	0
	Cerebral infarction, n (%)			28 (13.27%)	0
	Atrial fibrillation, n (%)			43 (20.38%)	0
	Myocardial infarction, n (%)			97 (45.97%)	0
Drug combination	Lipid-lowering drugs			85 (40.28%)	0
	Platelet-lowering drugs			65 (30.81%)	0
Essay index	RBC_admission, ×10^9/L, median (IQR)			4.13 (3.79–4.56)	1.90
	Hemoglobin_admission, g/L, median (IQR)			127.00 (115.00–139.00)	1.90
	WBC_admission, ×10^9/L, median (IQR)			7.80 (6.50–9.84)	2.84
	NEU_admission, ×10^9/L, median (IQR)			74.60 (62.80–83.60)	4.74
	Platelet_admission, ×10^9/L, median (IQR)			187.00 (147.50–232.50)	2.37
	HCT_admission, L/L, median (IQR)			37.50 (34.40–41.55)	1.90
	ALT_admission, U/L, median (IQR)			23.00 (13.25–36.00)	13.74
	AST_admission, U/L, median (IQR)			30.50 (20.25–77.75)	13.74
	GGT_admission, U/L, median (IQR)			25.50 (16.00–50.75)	39.34
	LDL_admission, g/L, median (IQR)			2.42 (1.71–3.03)	27.49
	Prealbumin_admission, g/L, median (IQR)			191.00 (143.00–237.00)	44.55
	BUN_admission, mmol/L, median (IQR)			5.90 (4.89–8.25)	13.27
	TBil_admission, μmol/L, median (IQR)			12.00 (9.60–17.65)	34.12
	TG_admission, mmol/L, median (IQR)			1.25 (0.82–1.77)	22.75
	Albumin_admission, g/L, median (IQR)			37.50 (34.30–40.60)	9.00
	DBil_admission, μmol/L, median (IQR)			3.50 (2.27–5.50)	35.55
	Cr_admission, μmol/L, median (IQR)			75.00 (63.00–97.00)	12.32
	Cholesterol_admission, mmol/L, median (IQR)			3.98 (3.24–4.93)	25.59
	Cl_admission, mmol/L, median (IQR)			103.40 (100.35–106.00)	39.81
	Na_admission, mmol/L, median (IQR)			138.00 (136.00–141.00)	39.81
	K_admission, mmol/L, median (IQR)			3.90 (3.60–4.11)	39.34
	HDLC_admission, mmol/L, median (IQR)			1.19 (1.03–1.41)	25.12
	PT_admission, minute, median (IQR)			12.75 (11.60–14.25)	7.11
	INR_admission, median (IQR)			1.09 (1.03–1.18)	7.11
	APTT_admission, minute, median (IQR)			31.25 (28.70–35.23)	7.11

Abbreviations: IQR, interquartile range; RBC, red blood cells; WBC, white blood cells; NEU, neutrophil; HCT, hematocrit; ALT; alanine transaminase; AST, aspartate aminotransferase; GGT, gamma-glutamyl transpeptidase; LDL, low density lipoprotein; BUN, blood urea nitrogen; TBil, total bilirubin; TG, total cholesterol; DBil, direct bilirubin; Cr, creatinine; HDLC, high density lipoprotein cholesterol; PT, prothrombin time; INR, international normalized ratio; APTT, activated partial thromboplastin time.

### 3.2 Analysis of clinical effect

According to prior knowledge and clinical experience, we found that age and comorbidities (such as myocardial infarction) were underlying factors in heart failure, significantly influencing its outcome and prognosis (Ziaeian and Fonarow, 2016). In addition, several large observational studies, *post hoc* analyses of RCTs, and small prospective trials have suggested that statins can be beneficial to patients with heart failure (Tavazzi et al., 2008). And for patients with chronic coronary artery disease or peripheral artery disease and heart failure, combination of rivaroxaban and aspirin compared with aspirin alone produced larger absolute benefits (Branch et al., 2019). Hereby, after considering the quality of data, the controlling variables in PSM were determined as age, lipid-lowering drugs, platelet-lowering drugs, and myocardial infarction. The dataset of the controlling variables before and after PSM was presented in Table 2. After PSM, the sample size of experimental group came into 106 cases, and 106 cases for the control group. After PSM, the median (IQR) age was 73.0 (67.3–81.0) years in the experimental group and 72.5 (66.0–81.0) years in the control group. The percentage of patients using lipid-lowering drugs was 42.5% in the experimental group and 44.3% in the control group. The percentage of patients using platelet-lowering drugs was 36.8% in the experimental group and 36.8% in the control group. The percentage of patients having myocardial infarction was 12.3% in the experimental group and 13.2% in the control group.

**TABLE 2:** Controlling variables before and after PSM.

	Before PSM				After PSM			
	Total (N = 487)	Experimental group (N = 211)	Control group (N = 276)	*p*-Value	Total (N = 212)	Experimental group (N = 106)	Control group (N = 106)	*p*-Value
Age, year								
Mean (SD)	71.5 (12.0)	71.0 (12.4)	71.8 (11.6)	0.514	73.1 (11.1)	73.6 (10.9)	72.6 (11.4)	0.572
Median (Q1∼Q3)	71.0 (63.0–81.0)	70.0 (63.0–81.0)	72.0 (63.8–81.0)		73.0 (66.0–81.0)	73.0 (67.3–81.0)	72.5 (66.0–81.0)	
Lipid-lowering drugs, N (%)								
0	225 (46.2%)	126 (59.7%)	99 (35.9%)	<0.001	120 (56.6%)	61 (57.5%)	59 (55.7%)	0.782
1	262 (53.8%)	85 (40.3%)	177 (64.1%)		92 (43.4%)	45 (42.5%)	47 (44.3%)	
Platelet-lowering drugs, N (%)								
0	274 (56.3%)	146 (69.2%)	128 (46.4%)	<0.001	134 (63.2%)	67 (63.2%)	67 (63.2%)	1
1	213 (43.7%)	65 (30.8%)	148 (53.6%)		78 (36.8%)	39 (36.8%)	39 (36.8%)	
Myocardial infarction, N (%)								
0	366 (75.2%)	114 (54.0%)	252 (91.3%)	<0.001	185 (87.3%)	93 (87.7%)	92 (86.8%)	0.837
1	121 (24.8%)	97 (46.0%)	24 (8.7%)		27 (12.7%)	13 (12.3%)	14 (13.2%)	

Notes: “No” corresponds to “0”, and “Yes” corresponds to “1”. Abbreviations: PSM, propensity score matching.

After matching, the normality test was conducted for the length of hospital stay between the experimental group and the control group, but the data distribution did not fit the normal distribution, thus Wilcoxon rank sum test was conducted to determine the statistical difference of the length of hospital stay between the experimental group and the control group. It shows in Table 3 that the median (IQR) length of hospital stay was 10.0 (8.0–14.0) days in the experimental group and 11.0 (10.0–14.0) days in the control group. Median difference (95%CI) was 1.0 (0.00003–1.99998) days. At the significance level of 0.05, there was a statistical difference in the length of hospital stay between the experimental group and the control group (*p* = 0.006).

**TABLE 3:** Comparison of length of hospital stay.

Length of hospital stay	Total (N = 212)	Experimental group (N = 106)	Control group (N = 106)	Median difference (95%CI)	*p*-value
Mean (SD)	12.9 (6.97)	12.8 (8.51)	12.9 (5.02)	1.0 (0.00003–1.99998)	0.006
Median (IQR)	11.0 (9.0–14.0)	10.0 (8.00–14.0)	11.0 (10.0–14.0)		

Abbreviations: SD, standard deviation; IQR, interquartile range; CI, confidence interval.

### 3.3 Variable analysis

**FIGURE 3:**
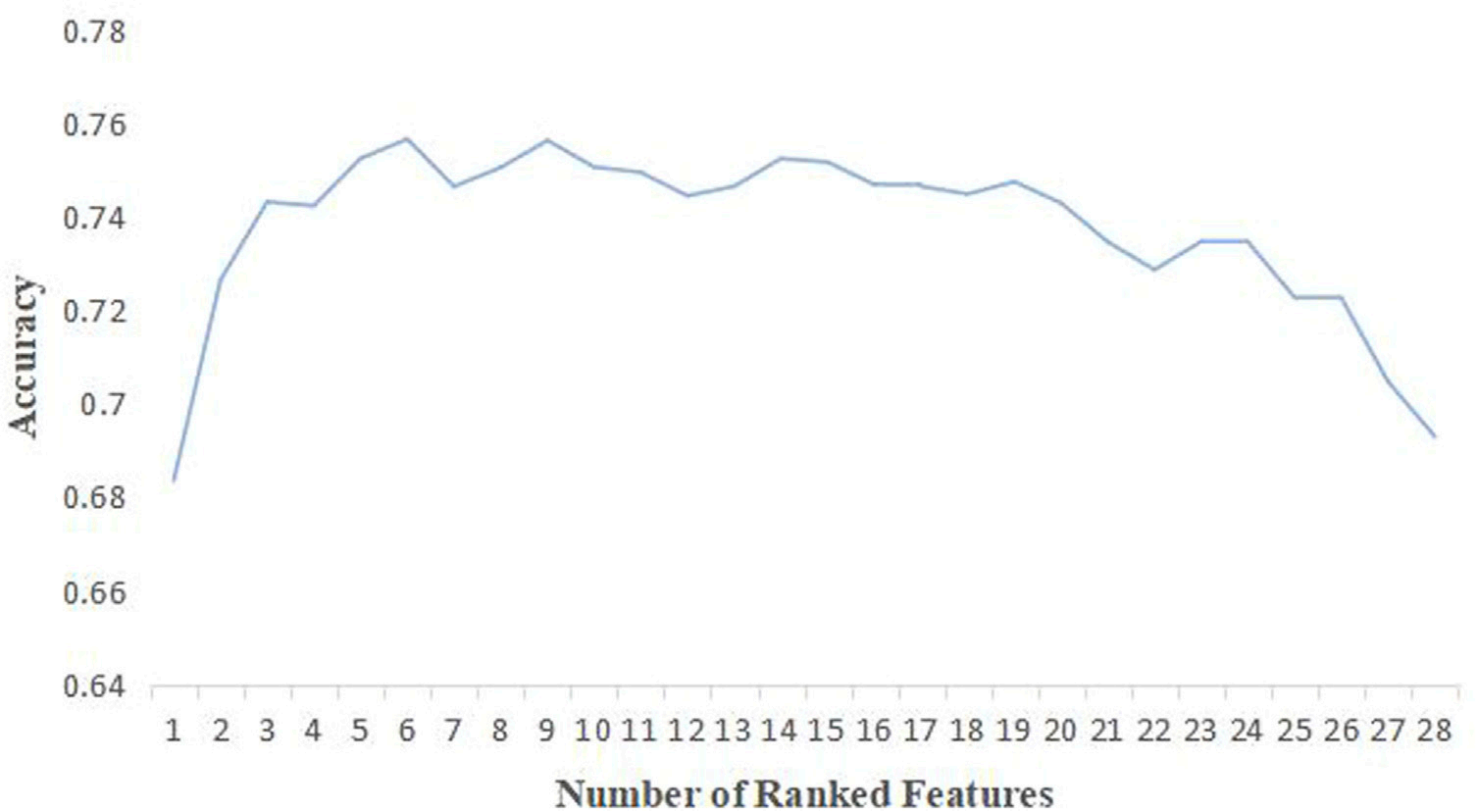
Accuracy of XGBoost model corresponding to the number of ranked features.

After data preprocessing, according to significance test results and medical relevance, 9 variables with *p*-value greater than 0.05 were removed from the medication data set, and 28 variables were left. Then, features were selected based on 28 variables through SFS method. XGBoost models were established using the selected 1 to 28 variables, and the accuracy of each model was obtained (Figure 3). With increasing number of included variables, the value of accuracy kept rising, reached its maximum value when six features were selected (accuracy = 0.75) and then declined. As we pursued a concise and accurate model with minimal variables but highest predictive performance, the first six variables were selected to establish the personalized medicine model, including gamma-glutamyl transpeptidase (GGT)_admission, lipid-lowering drugs, hemoglobin_admission, prealbumin_admission, cholesterol_admission, and platelet-lowering drugs.

### 3.4 Model performance

The variables screened out above, lipid-lowering drugs, platelet-lowering drugs, levels of GGT, hemoglobin, prealbumin, and cholesterol at admission, were used as covariables in establishing prediction models. The predictive performance of nine models in the test cohort after six-fold cross validation was displayed in Table 4. We can see that XGBoost model had precision of 0.77 ± 0.15, recall of 0.78 ± 0.09, F1_score of 0.76 ± 0.05 for predicting daily dose of 40 mg Shengmai injection; precision of 0.84 ± 0.11, recall of 0.85 ± 0.1, F1_score of 0.84 ± 0.04 for predicting daily dose of 60 mg Shengmai injection; and accuracy of 0.81 ± 0.03, AUC of 0.87 ± 0.02, sensitivity of 0.85 ± 0.1, and specificity of 0.78 ± 0.09 for the whole XGBoost model, the metrics of which were higher than other algorithms and achieved a best comprehensive performance. The comparison of baseline data between the original and external cohorts indicates no considerable differences of the baseline data basically exist between two cohorts (Supplementary Table S1). In external verification cohort, as shown in Table 5, XGBoost model had precision of 0.80, recall of 0.76, F1_score of 0.78 for predicting daily dose of 40 mg Shengmai injection; precision of 0.87, recall of 0.89, F1_score of 0.88 for predicting daily dose of 60 mg Shengmai injection; and accuracy of 0.84, AUC of 0.84, sensitivity of 0.89, and specificity of 0.76 for the whole model. Therefore, XGBoost was selected to predict daily dose of Shengmai injection, and to calculate the importance scores of variables and analyze the dose prediction effect.

**TABLE 4:** Prediction performance of different algorithms in the test cohort.

Model	Label	Precision	Recall	F1	Accuracy	AUC	Sensitivity	Specificity
XGBoost	0	0.77 ± 0.15	0.78 ± 0.09	0.76 ± 0.05	0.81 ± 0.03	0.87 ± 0.02	0.85 ± 0.1	0.78 ± 0.09
	1	0.84 ± 0.11	0.85 ± 0.1	0.84 ± 0.04				
LightGBM	0	0.71 ± 0.15	0.7 ± 0.09	0.7 ± 0.05	0.77 ± 0.03	0.84 ± 0.02	0.82 ± 0.1	0.7 ± 0.09
	1	0.8 ± 0.11	0.82 ± 0.1	0.81 ± 0.04				
CatBoost	0	0.68 ± 0.15	0.65 ± 0.09	0.65 ± 0.05	0.73 ± 0.03	0.79 ± 0.02	0.8 ± 0.1	0.65 ± 0.09
	1	0.76 ± 0.11	0.8 ± 0.1	0.77 ± 0.04				
RF	0	0.72 ± 0.15	0.68 ± 0.09	0.68 ± 0.05	0.76 ± 0.03	0.85 ± 0.02	0.83 ± 0.1	0.68 ± 0.09
	1	0.78 ± 0.11	0.83 ± 0.1	0.8 ± 0.04				
GBDT	0	0.71 ± 0.15	0.7 ± 0.09	0.68 ± 0.05	0.76 ± 0.03	0.82 ± 0.02	0.82 ± 0.1	0.7 ± 0.09
	1	0.8 ± 0.11	0.82 ± 0.1	0.8 ± 0.04				
SVR	0	0.62 ± 0.15	0.61 ± 0.09	0.57 ± 0.05	0.67 ± 0.03	0.79 ± 0.02	0.76 ± 0.1	0.61 ± 0.09
	1	0.74 ± 0.11	0.76 ± 0.1	0.73 ± 0.04				
LR	0	0.7 ± 0.15	0.68 ± 0.09	0.67 ± 0.05	0.74 ± 0.03	0.82 ± 0.02	0.81 ± 0.1	0.68 ± 0.09
	1	0.78 ± 0.11	0.81 ± 0.1	0.78 ± 0.04				
ANN	0	0.65 ± 0.15	0.34 ± 0.09	0.37 ± 0.05	0.64 ± 0.03	0.64 ± 0.02	0.9 ± 0.1	0.34 ± 0.09
	1	0.66 ± 0.11	0.9 ± 0.1	0.74 ± 0.04				
TabNet	0	0.62 ± 0.15	0.61 ± 0.09	0.57 ± 0.05	0.67 ± 0.03	0.79 ± 0.02	0.76 ± 0.1	0.61 ± 0.09
	1	0.74 ± 0.11	0.76 ± 0.1	0.73 ± 0.04				

Notes: Regimen of daily dose of 40 mg Shengmai injection corresponds to “0”, and regimen of daily dose of 60 mg Shengmai injection corresponds to “1”. Abbreviations: XGBoost, eXtreme Gradient Boosting; CatBoost, Categorical Boosting; RF, random forest; GBDT, gradient boosting decision tree; SVR, support vector regression; LR, logistic regression; ANN, artificial neural network; LightGBM, light gradient boosting machine.

**TABLE 5:** Prediction performance of different algorithms in the external verification cohort.

Model	Label	Precision	Recall	F1	Accuracy	AUC	Sensitivity	Specificity
XGBoost	0	0.80	0.76	0.78	0.84	0.84	0.89	0.76
	1	0.87	0.89	0.88				
LightGBM	0	0.64	0.67	0.65	0.74	0.76	0.78	0.67
	1	0.81	0.78	0.79				
CatBoost	0	0.56	0.71	0.63	0.69	0.73	0.68	0.71
	1	0.81	0.68	0.74				
RF	0	0.65	0.52	0.58	0.72	0.71	0.84	0.52
	1	0.76	0.84	0.79				
GBDT	0	0.58	0.71	0.64	0.71	0.72	0.70	0.71
	1	0.81	0.70	0.75				
SVR	0	0.40	0.38	0.39	0.57	0.58	0.68	0.38
	1	0.66	0.68	0.67				
LR	0	0.62	0.38	0.47	0.69	0.71	0.68	0.38
	1	0.71	0.86	0.78				
ANN	0	0.50	0.33	0.40	0.64	0.72	0.81	0.33
	1	0.68	0.81	0.74				
TabNet	0	0.58	0.71	0.64	0.71	0.72	0.70	0.71
	1	0.81	0.70	0.75				

Notes: Regimen of daily dose of 40 mg Shengmai injection corresponds to “0”, and regimen of daily dose of 60 mg Shengmai injection corresponds to “1”. Abbreviations: XGBoost, eXtreme Gradient Boosting; CatBoost, Categorical Boosting; RF, random forest; GBDT, gradient boosting decision tree; SVR, support vector regression; LR, logistic regression; ANN, artificial neural network; LightGBM, light gradient boosting machine.

### 3.5 Clinical interpretation

On this basis, the importance scores of six selected variables were calculated and ranked by XGBoost after cross validation (Table 6). Among them, the importance score of hemoglobin_admission was higher than other variables (importance score = 0.21 ± 0.01), followed by cholesterol_admission (importance score = 0.19 ± 0.02), prealbumin_admission (importance score = 0.17 ± 0.01), platelet-lowering drugs (importance score = 0.15 ± 0.02), GGT_admission (importance score = 0.14 ± 0.01), and lipid-lowering drugs (importance score = 0.13 ± 0.02). The higher score indicates the larger impact of this variable on predicting the daily dose of Shengmai injection.

**TABLE 6:** Importance score of variables.

Rank	Variable	Importance score
1	Hemoglobin_admission	0.21 ± 0.01
2	Cholesterol_admission	0.19 ± 0.02
3	Prealbumin_admission	0.17 ± 0.01
4	Platelet lowering drugs	0.15 ± 0.02
5	GGT_admission	0.14 ± 0.01
6	Lipid-lowering drugs	0.13 ± 0.02

Abbreviations: GGT, gamma-glutamyl transpeptidase.

**FIGURE 4:**
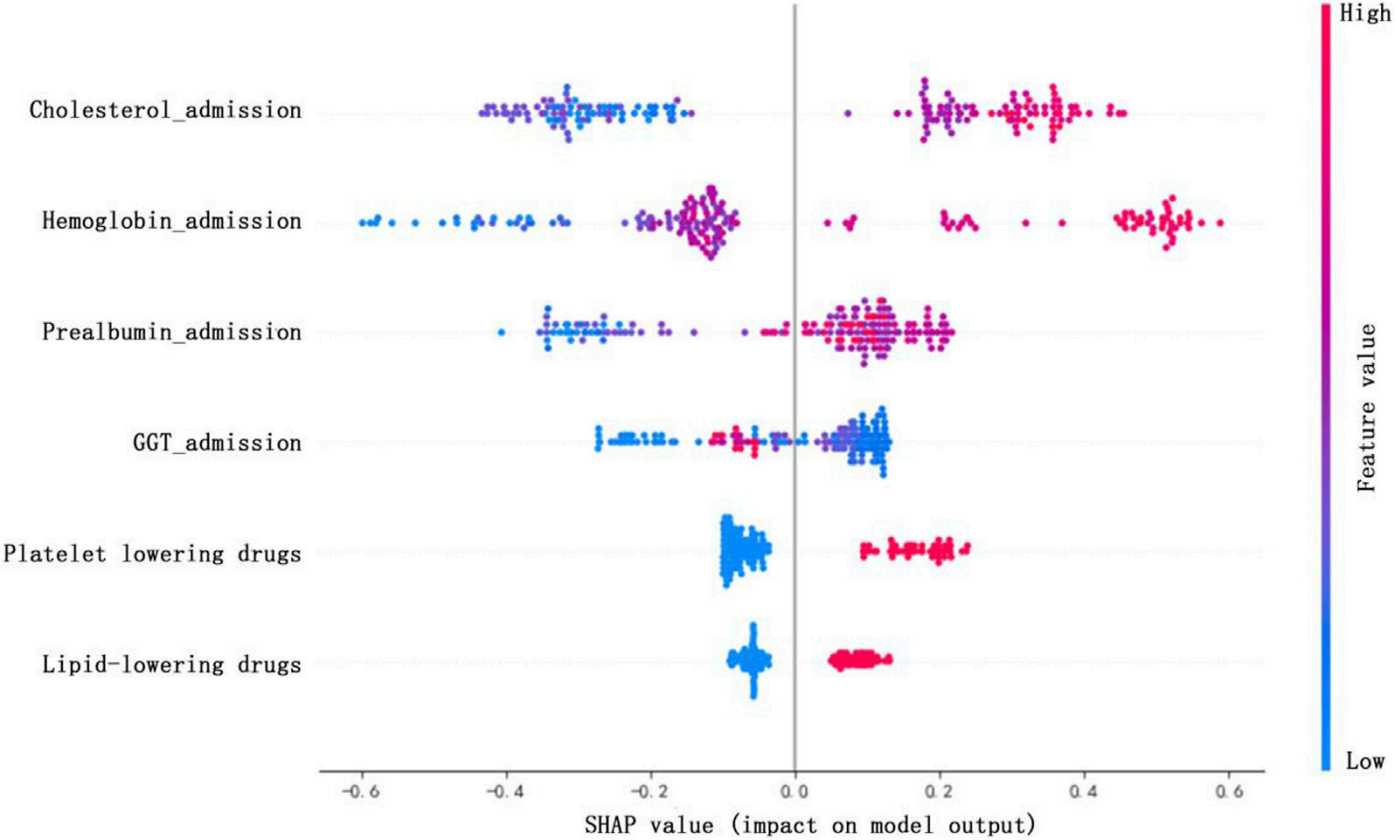
SHAP values of the important variables. The dot color is redder when the feature value gets higher and bluer when the feature value gets lower. When the SHAP value gets higher, the impact of the variable on model output is larger.

In the XGBoost model, SHAP values were used to show the distribution of each variable’s impact on the model output (Figure 4). It can be seen that variables including platelet-lowering drugs, lipid-lowering drugs, cholesterol_admission, hemoglobin_admission and prealbumin_admission show positive impacts on the daily dose of Shengmai injection. Whereas GGT_admission shows a negative correlation with the daily dose of Shengmai injection.

**FIGURE 5:**
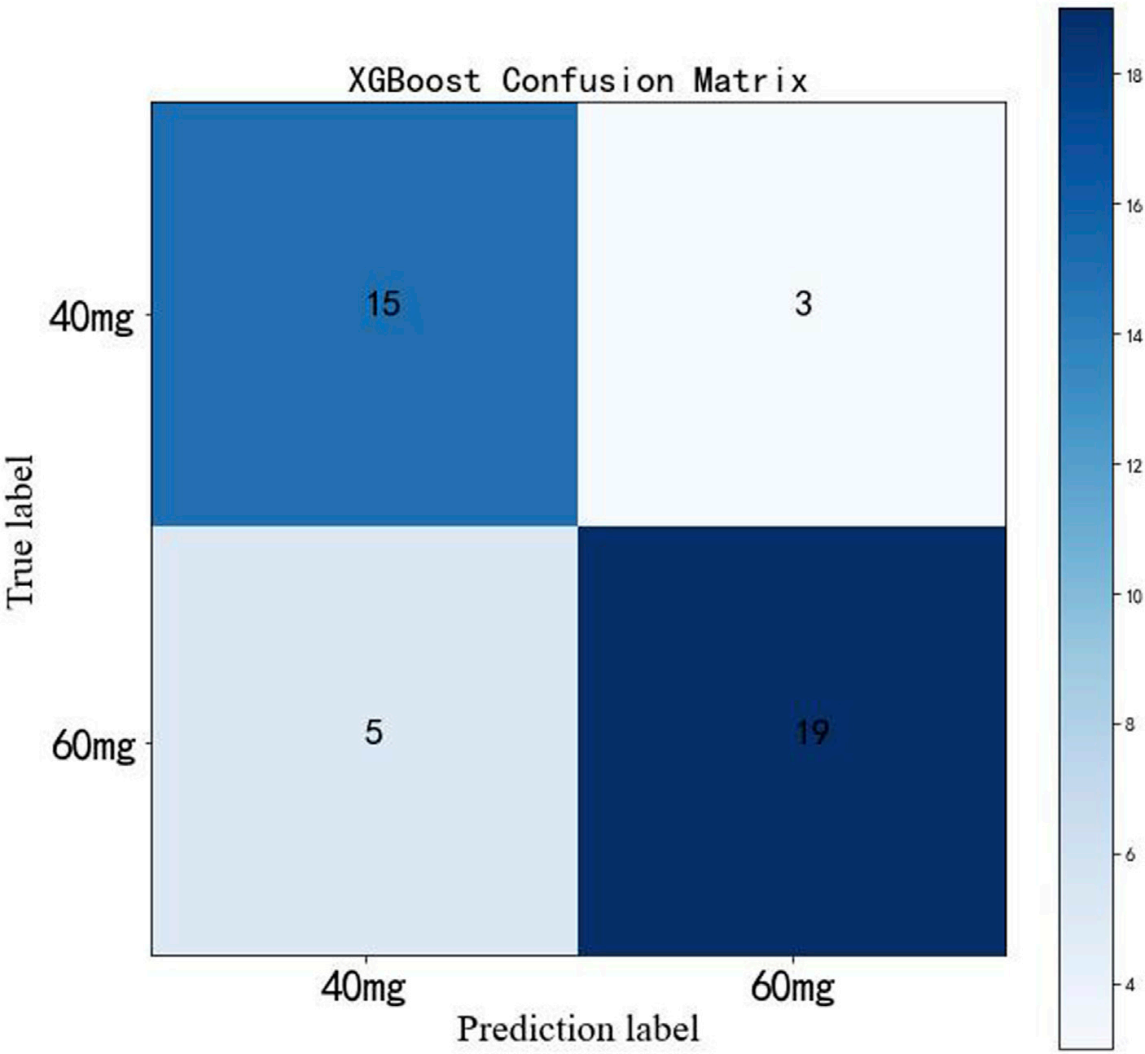
Confusion matrix in XGBoost model.

The test cohort consisted of 42 patients with coronary heart disease, 18 patients of which took daily dose of 40 mg Shengmai injection and 24 patients took daily dose of 60 mg Shengmai injection. The dose of Shengmai injection was predicted for patients by establishing confusion matrix based on XGBoost prediction model (Figure 5). The model predicted daily dose of 40 mg Shengmai injection accurately for 15 patients, with the precision of 75%, recall rate of 83% and F1-score of 79%; the model predicted daily dose of 60 mg Shengmai injection accurately for 19 patients, with the precision of 86%, recall rate of 79% and F1-score of 83%.

## 4 Discussion

Coronary heart disease, which includes heart failure, arrhythmias, acute coronary syndromes (such as myocardial infarction and unstable angina), and sudden cardiac death, is a major cause of morbidity and mortality globally, leading to a heavy burden on economy and health (Townsend et al., 2016; Benjamin et al., 2017; Wirtz and von Känel, 2017). Currently, Shengmai injection is used as a complementary treatment to Western medication treatments in China, usually recommended in treating heart failure in clinics (Zhang and Liu, 2013; Zhou et al., 2014). However, evaluation of clinical effectiveness and safety of Shengmai injection is lacking. In this study, we aimed to explore the clinical effect of Shengmai injection through the length of hospital stay, and establish a personalized medicine model of Shengmai injection to achieve optimal therapeutic regimen.

The median length of hospital stay of the experimental group was shorter than the control group (10.00 days vs. 11.00 days, *p* = 0.006), which indicates that Shengmai injection can considerably reduce the length of hospital stay in patients with coronary heart disease. A meta-analysis of some RCTs about the effectiveness of Shengmai injection shows that cardiac function indicators (including left ventricular ejection fraction, stroke volume, cardiac output, and cardiac index) were usually chosen as the effectiveness evaluation indexes (Wang et al., 2020b). However, their outcomes had a high heterogeneity (Wang et al., 2020b). In this study, the reduction of the length of hospital stay after using Shengmai injection could be attributed to the improvement of cardiac function, infarct area, myocardial damage and ventricular hypertrophy (Chen et al., 2013; Qu et al., 2022). In further analysis, more clinical evaluation indexes relevant to effectiveness should be considered and long term follow-up should be performed.

During the establishment of personalized medicine model, hemoglobin_admission, cholesterol_admission, prealbumin_admission, and GGT_admission were identified as the important variables considerably influencing the dose regimen of Shengmai injection. Hemoglobin level can reflect the state of blood. Li WN et al. found that the incidence and severity of anemia, which was indicated by the low hemoglobin level, were positively correlated with the severity of chronic heart failure (Wen-Ni et al., 2017). Meanwhile, high level of hemoglobin was proven to negatively affect the prognosis of coronary heart disease, since it promoted the progression of atherosclerosis and thrombosis (Quan et al., 2013). Thus, hemoglobin level tested at admission can indirectly reflect the severity of coronary heart disease, which may remarkably influence the medication regimen for treatment. In addition, abnormal cholesterol level (increased total cholesterol) was a pathogenic factor in atherosclerosis (Zárate et al., 2016). Appropriate dose of Shengmai treatment could regulate the lipid metabolism disorders, reduce plaque area, increase collagen fiber content, and reduce the risk of plaque rupture in previous study, since ginsenoside played an important role in maintaining the homeostasis of lipids, such as cholesterol and lysophosphatidyl choline (Wang et al., 2021; Qu et al., 2022). Hence, high cholesterol level at admission indicated greater incidence of coronary atherosclerotic events, and it is important to consider the cholesterol level at admission when developing Shengmai regimen. As a non-specific host defense substance, prealbumin, whose level was associated with atherosclerosis and represented the severity of coronary heart disease, was gradually consumed during the removal of toxic metabolites (Ren and Li, 2013; Chen and Wei, 2008; Yan et al., 2014). The concentration of serum prealbumin decreased gradually with the aggravation of the coronary heart disease, because of the inflammatory lesions in the development of atherosclerosis (Yan et al.). Decreased level of prealbumin indicated severe coronary heart disease, which may imply high-dose regimen of Shengmai treatment. However, SHAP values display that elevated level of prealbumin at admission may be inclined to use high-dose regimen of Shengmai injection. We supposed that the inconsistent outcome may be due to the biased data caused by a high missing rate (44.55%). Regarding to GGT level, we found it had a weak negative correlation with the daily dose of Shengmai injection, oppositely to the outcomes of previous studies. Zhang F et al. proved a positive correlation between GGT and Gensini score (a score criterion to evaluate the severity of coronary atherosclerosis and higher score indicates severer coronary stenosis), and elevated GGT was an independent risk factor associated with coronary heart disease (Zhang et al., 2022). Consistent with this, some studies found that a large amount of GGT existed in the intimal plaques, and positively correlated with the severity of the lesions (Shabbir et al., 2011; Aksakal et al., 2012). High GGT level may accumulate activated GGT enzymes in atherosclerotic plaques, mediate various oxidative reactions, and affect plaque progression and stability (Zhang et al.). As a result, elevated GGT level tended to indicate severe coronary heart disease and may imply high-dose regimen of Shengmai injection, but our outcome shows the inverse relationship. In our study, the inconsistent outcome may be due to the biased data caused by a high missing rate (39.34%).

Moreover, some combined drugs were also identified as the important variables influencing the dose regimen of Shengmai injection, such as platelet-lowering drugs and lipid-lowering drugs. Commonly, platelet-lowering drugs and lipid-lowering drugs were used as the mainstay treatment for coronary heart disease. For instance, aspirin was normally used as routine antiplatelet aggregation therapy in clinics and showed positive effects on reducing the expression of inflammatory cytokines, antiplatelet, antiatherosclerosis, and preventing thrombosis (Mao et al., 2021). Similarly, lipid-lowering therapy was mainly used to reduce cardiovascular events. For example, statins were capable to reduce morbidity and mortality of cardiovascular disease remarkably through cholesterol reduction, inflammation reduction, vascular tone improvement, and platelet aggregation reduction (Almeida and Budoff, 2019). In clinic, we can deduce that patients using platelet-lowering drugs and/or lipid-lowering drugs as combined treatments had severer conditions of coronary heart disease, which may influence the treatment regimen of Shengmai injection.

In this work, SHAP values display that platelet-lowering drugs, lipid-lowering drugs, cholesterol_admission, hemoglobin_admission and prealbumin_admission have positive correlations with the daily dose of Shengmai injection, patients with elevated levels of cholesterol and/or hemoglobin and/or prealbumin at admission, and/or using platelet-lowering drugs and/or lipid-lowering drugs are apt to use 60 mg regimen of Shengmai injection. Additionally, GGT_admission shows a negative correlation with the daily dose of Shengmai injection, thus patients with decreased level of GGT are apt to use 40 mg regimen of Shengmai injection. Due to some variables with high rate of missing values, further research is needed to validate the results. According to the confusion matrix results, the classifier correctly identified 75% of patients using 40 mg regimen of Shengmai injection and 86% of patients using 60 mg regimen of Shengmai injection, indicating a good prediction performance. Nevertheless, the sample size in this study was small, which needs large samples to verify this result in future.

In this study, we screened important variables associated with the dose of Shengmai injection and established a personalized medicine model via a powerful machine learning technique, XGBoost. XGBoost is a machine learning algorithm on the basis of the upgrade of the GBDT algorithm, which can integrate multiple decision trees to achieve regression or classification goals (Chen and Guestrin, 2016). The decision tree is simple and easy to understand, but it has great risk of over-fitting and limited application scenarios. The random forest adopts bagging sampling, random attribute selection and model integration to solve the risk of over-fitting in decision tree, but at the expense of interpretability. On the basis of random forest, GBDT integrated boosting to establish the connection between trees, which makes the forest no longer exist as independent trees, and then become an ordered collective decision-making system. XGBoost goes one step further on the basis of GBDT, adding regular terms into the objective function of each iteration to further reduce the risk of overfitting. Compared with the GBDT heuristic iterative principle, XGBoost’s optimization criterion is completely based on the minimization of the objective function and adopts the second-order Taylor expansion to make it possible to define the loss function. The advantages of XGBoost lead it outperform other algorithms. Specifically, it can process data rapidly and effectively. In order to reduce overfitting and control the model complexity, it supports parallel computing, column sampling and incorporates regularization (Chen and Guestrin, 2016). Built-in rules and cross-validation were used for dealing with missing values and improving model stability (Chen and Guestrin, 2016). XGBoost is robust to highly correlated variables and sparse matrix (Chen and Guestrin, 2016). In this study, XGBoost performs better than other algorithms might because of its better processing capabilities for clinical data with a mass of outliers and missing values (Zhang et al., 2020). The capacity of XGBoost has been shown in some previous studies. For instance, Huang X et al. established a dose prediction model of vancomycin through XGBoost, which helps to deeply explore second-order variable interactions to enhance the model performance (Huang et al., 2021). Machine learning models always show powerful predictive abilities, since they are adept at processing high-dimensional data as well as the non-linear relationships between predictors and objectives, and these models have good scalability. It means that the prediction model can be updated through the automatic extraction of data from electronic health records and continuous monitor of physiological data.

In the evaluation of models, accuracy is a simple and intuitive evaluation indicator for classification problems, but accuracy can be affected by the data categories with large sample size, when the sample data are extremely unbalanced. Precision and recall are both contradictory and uniform indicators. F1 score is the harmonic mean of precision and recall, and F1 score can find a balance between the two indicators to reach their maximum value. ROC can comprehensively reflect the performance of a sorting model. Sensitivity and specificity are not affected by the unbalanced sample data. Each evaluation indicator has its value and applicable situation, whereas evaluation with a single indicator will draw a one-sided or even wrong conclusion. Only through a set of complementary evaluation indicators, the problems of the model can be found and solved. Therefore, Precision, recall, F1, accuracy, AUC, sensitivity and specificity were adopted in this study to comprehensively evaluate the model performance, and the results indicate a good performance. The unbalanced data in this study have been removed, weakening the corresponding impact.

RCT is generally considered the gold standard for evaluating drug safety and efficacy. However, rigorous enrollment conditions and quality control of clinical procedures lead to difficulty to reproduce RCT efficacy evaluation results in real medical situations, weakening the relevance of RCT findings to the real world (Kim et al., 2018). While RCT focuses on drug efficacy, real-world study concentrates on drug effectiveness in real clinical settings (Kim et al., 2018). We can say that RCT and real-world study are mutually complementary. In our study, we explored the clinical effect of Shengmai based on real-world data, and after finding that it positively affected the length of hospital stay, we established a personalized medicine model, expecting to provide reference for the treatment regimen of patients. In the future, we aimed to establish a multicenter RCT in patients treated with shengmai injection. Patients would be randomly divided into model-informed dosing group and standard dosing group. The main outcome measures would be the length of hospital stay, left ventricular ejection fraction, grade of NYHA and other clinical evaluation indexes. An independent sample *t*-test and chi square test would be used to test the statistical significance between two groups. The outcome of RCT can complement powerful evidence for the model performance in this study.

Some limitations existed in this study. One is the limited sample size from one single center. Our findings need to be verified by larger sample size, double-blind, multi-center research. Secondly, some variables have high missing rates, inevitable in real-world study, which may lead to biased outcome. Some statistical models can be used for missing filling in future research, such as multiple imputation, fully conditional specification, and Markov Chain Monte Carlo. Thirdly, real-world study has noticeable demerits. It is non-experimental, leading to inevitable interference of various confounding factors. We applied PSM to correct for confounding bias, but the available sample size was substantially reduced after matching. In this study, we chose the controlling variables on the basis of clinical experience and prior knowledge, and some underlying confounding factors were not analyzed due to limited dataset or unfulfilled data inclusion criteria, such as the using duration of Shengmai, quality of raw materials, processing technology, drug combination of β-blocker or nitrates, and complex clinical situations including severity of illness, multiple complications, malnutrition or immune deficiency. Even we controlled certain confounding factors that may interfere the outcome, there still existed some unknown confounders from real world. In future study, we plan to apply a RCT with rigorous inclusion criteria to control underlying confounders. Based on the principle of randomization, the subjects are assigned to each group with the same probability, thus the potential confounding variables can be evenly distributed among the groups. Besides, PSM, multiple regression and stratified analysis were recommended for reducing bias.

## 5 Conclusion

In conclusion, we conducted a real-world study about Shengmai injection. Firstly, we found that patients using Shengmai injection had shorter length of hospital stay than those not using Shengmai injection, which is beneficial to the save of cost and medical resource for both patients and hospitals. Secondly, we established a personalized medicine model of Shengmai injection by XGBoost. The important variables for establishing the personalized medicine model of Shengmai injection included lipid-lowering drugs, platelet-lowering drugs, levels of GGT, hemoglobin, prealbumin, and cholesterol at admission. This study proves the benefit of using Shengmai injection in patients with coronary heart disease via reducing medical burden and cost, and illustrated a personalized medicine model to accurately predict dose regimen of Shengmai injection, which is expected to be applied in practical application and help doctors make better clinical decisions to achieve an optimum of effectiveness and safety. It also provides reference of using real-world data for complementing evidence of drug evaluation besides RCT.

## Data Availability

The raw data supporting the conclusion of this article will be made available by the authors, without undue reservation.
