# Supplementary material for: The effect of Shengmai injection in patients with coronary heart disease in real world and its personalized medicine research using machine learning techniques
Source: Front Pharmacol. 2023 Sep 14;14:1208621. doi: 10.3389/fphar.2023.1208621 (PMC10537936; doi:10.3389/fphar.2023.1208621)
Supplement: Supplementary file 1 [file Table1.DOCX]

**Supplementary Table S1**

**Table S1. Comparison of baseline information between original cohort and external verification cohort**

| **Categories** | **Variables** | | | **Original cohort** | | **External verification cohort** | | **P value** |
| --- | --- | --- | --- | --- | --- | --- | --- | --- |
|  |  |  |  | **Cases (N=211)** | **Missing rate** | **Cases (N=58)** | **Missing rate** |  |
| Target variable | Daily dose of Shengmai injection, mg, median (IQR) | | 40mg | 95(45.02%) | 0% | 21(36.21%) | 0% | 1.0 |
|  |  |  | 60mg | 116(54.98%) | 0% | 37(63.79%) | 0% |  |
| Medication information | Medication days, d, median (IQR) | | | 6.00(4.00-8.00) | 0% | 5.00(3.00-6.75) | 0% | 0.059 |
|  | Length of hospital stay, d, median (IQR) | | | 11.00(9.00-16.00) | 0% | 9.00(7.00-12.00) | 0% | 0.002 |
| Demographic information | Age, y, median (IQR) | | | 70.00(63.00-81.00) | 0% | 73.00(67.00-83.00) | 0% | 0.122 |
|  | Gender, n (%) | Male | | 137(64.93%) | 0% | 37(63.79%) | 0% | 1.0 |
|  |  | Female | | 74(35.07%) | 0% | 21(36.21%) | 0% |  |
| Basic disease | Basic disease, n (%) | | | 168(79.62%) | 0% | 58(100.00%) | 0% | 1.0 |
|  | Coronary heart disease, n (%) | | | 195(92.42%) | 0% | 36(62.07%) | 0% | 0.897 |
|  | Pulmonary infection, n (%) | | | 42(19.91%) | 0% | 21(36.21%) | 0% | 0.234 |
|  | Cerebral infarction, n (%) | | | 28(13.27%) | 0% | 21(36.21%) | 0% | 1.0 |
|  | Atrial fibrillation, n (%) | | | 43(20.38%) | 0% | 13(22.41%) | 0% | 0.670 |
|  | Myocardial infarction, n (%) | | | 97(45.97%) | 0% | 27(46.55%) | 0% | 0.163 |
| Drug combination | Lipid-lowering drugs | | | 85(40.28%) | 0% | 51(87.93%) | 0% | 1.0 |
|  | Platelet-lowering drugs | | | 65(30.81%) | 0% | 49(84.48%) | 0% | 0.670 |
| Essay index | RBC_admission, ×10^9/L, median (IQR) | | | 4.13(3.79-4.56) | 1.90% | 4.14(3.87-4.58) | 1.72% | 0.598 |
|  | Hemoglobin_admission, g/L, median (IQR) | | | 127.00(115.00-139.00) | 1.90% | 61.36(31.36-102.57) | 1.72% | 0.0 |
|  | WBC_admission, ×10^9/L, median (IQR) | | | 7.80(6.50-9.84) | 2.84% | 7.50(5.70-8.79) | 1.72% | 0.098 |
|  | NEU_admission, ×10^9/L, median (IQR) | | | 74.60(62.80-83.60) | 4.74% | 69.00(61.90-80.10) | 1.72% | 0.107 |
|  | Platelet_admission, ×10^9/L, median (IQR) | | | 187.00(147.50-232.50) | 2.37% | 188.00(149.00-225.00) | 1.72% | 0.819 |
|  | HCT_admission, L/L, median (IQR) | | | 37.50(34.40-41.55) | 1.90% | 37.20(34.20-41.50) | 1.72% | 0.643 |
|  | ALT_admission, U/L, median (IQR) | | | 23.00(13.25-36.00) | 13.74% | 26.00(20.00-39.00) | 36.21% | 0.187 |
|  | AST_admission, U/L, median (IQR) | | | 30.50(20.25-77.75) | 13.74% | 30.50(20.25-59.25) | 48.28% | 0.889 |
|  | GGT_admission, U/L, median (IQR) | | | 25.50(16.00-50.75) | 39.34% | 51.81(34.61-57.96) | 50.00% | 0.003 |
|  | LDL_admission, g/L, median (IQR) | | | 2.42(1.71-3.03) | 27.49% | 2.10(1.78-2.91) | 58.62% | 0.472 |
|  | Prealbumin_admission, g/L, median (IQR) | | | 191.00(143.00-237.00) | 44.55% | 56.92(40.87-135.68) | 60.34% | 0.0 |
|  | BUN_admission, mmol/L, median (IQR) | | | 5.90(4.89-8.25) | 13.27% | 7.00(5.00-9.29) | 27.59% | 0.319 |
|  | TBil_admission, μmol/L, median (IQR) | | | 12.00(9.60-17.65) | 34.12% | 13.60(10.40-19.10) | 36.21% | 0.167 |
|  | TG_admission, mmol/L, median (IQR) | | | 1.25(0.82-1.77) | 22.75% | 1.16(0.91-1.66) | 58.62% | 0.872 |
|  | Albumin_admission, g/L, median (IQR) | | | 37.50(34.30-40.60) | 9.00% | 39.65(37.30-42.10) | 34.48% | 0.013 |
|  | DBil_admission, μmol/L, median (IQR) | | | 3.50(2.27-5.50) | 35.55% | 3.50(2.40-4.55) | 53.45% | 0.841 |
|  | Cr_admission, μmol/L, median (IQR) | | | 75.00(63.00-97.00) | 12.32% | 80.00(61.00-102.00) | 29.31% | 0.831 |
|  | Cholesterol_admission, mmol/L, median (IQR) | | | 3.98(3.24-4.93) | 25.59% | 5.79(3.37-11.74) | 58.62% | 0.009 |
|  | Cl_admission, mmol/L, median (IQR) | | | 103.40(100.35-106.00) | 39.81% | 105.00(102.00-107.00) | 1.72% | 0.116 |
|  | Na_admission, mmol/L, median (IQR) | | | 138.00(136.00-141.00) | 39.81% | 140.00(138.00-142.00) | 3.45% | 0.005 |
|  | K_admission, mmol/L, median (IQR) | | | 3.90(3.60-4.11) | 39.34% | 3.90(3.60-4.10) | 5.17% | 0.914 |
|  | HDLC_admission, mmol/L, median (IQR) | | | 1.19(1.03-1.41) | 25.12% | 1.19(1.07-1.44) | 58.62% | 0.52 |
|  | PT_admission, minute, median (IQR) | | | 12.75(11.60-14.25) | 7.11% | 12.40(11.57-13.03) | 3.45% | 0.092 |
|  | INR_admission, median (IQR) | | | 1.09(1.03-1.18) | 7.11% | 1.12(1.05-1.18) | 3.45% | 0.302 |
|  | APTT_admission, minute, median (IQR) | | | 31.25(28.70-35.23) | 7.11% | 30.70(28.77-34.73) | 3.45% | 0.746 |

Abbreviations: IQR, interquartile range; RBC, red blood cells; WBC, white blood cells; NEU, neutrophil; HCT, hematocrit; ALT; alanine transaminase; AST, aspartate aminotransferase; GGT, gamma-glutamyl transpeptidase; LDL, low density lipoprotein; BUN, blood urea nitrogen; TBil, total bilirubin; TG, total cholesterol; DBil, direct bilirubin; Cr, creatinine; HDLC, high density lipoprotein cholesterol; PT, prothrombin time; INR, international normalized ratio; APTT, activated partial thromboplastin time.
